# Inhibition of ox‐LDL‐induced endothelial cell injury by LINC02381 knockdown through the microRNA‐491‐5p/transcription factor 7 axis

**DOI:** 10.1002/iid3.785

**Published:** 2023-03-14

**Authors:** Xizheng Zhu, Hui Xu, Beijia Chen

**Affiliations:** ^1^ Department of Interventional Radiology Wuhan Asia General Hospital Wuhan China; ^2^ Department of Cardiology Fifth Hospital in Wuhan Wuhan China

**Keywords:** atherosclerosis, lncRNA, lncRNA LINC02381, miR‐491‐5p, miRNA

## Abstract

Atherosclerosis (AS) is a complex multifactorial and chronic inflammatory vascular disease that contributes to the development of cardiovascular diseases. Abnormal cellular proliferation in human umbilical vein endothelial cells (HUVECs) is a crucial element in AS development. In this study, we investigated the potential role of the long noncoding RNA LINC02381/microRNA (miR)‐491‐5p/transcription factor 7 (TCF7) axis in regulating HUVEC injury in 30 participants suffering from AS and 30 healthy control participants. We established an in vitro model of AS in HUVECs using oxidized low‐density lipoprotein (ox‐LDL), and measured cellular mRNA and protein levels of LINC02381, miR‐491‐5p, and TCF7 in serum samples using reverse transcription‐quantitative polymerase chain reaction and Western blotting assays. We evaluated cell viability, apoptosis, and inflammation using Cell Counting Kit‐8, flow cytometry, and enzyme‐linked immunosorbent assays, respectively. Moreover, we analyzed apoptosis‐related protein expression using western blotting analysis and determined the association between miR‐491‐5p and LINC02381 or TCF7 using dual‐luciferase reporter assay, RNA pull‐down, and rescue experiments. We observed that LINC02381 was elevated, while miR‐491‐5p was downregulated in serum samples from participants with AS and in ox‐LDL‐treated HUVECs. LINC02381 knockdown was protective against HUVEC injury via miR‐491‐5p inhibition, which is its downstream target. Rescue experiments further demonstrated that miR‐491‐5p alleviated HUVEC injury by modulating TCF7. Thus, LINC02381 knockdown ameliorated HUVEC injury by regulating the miR‐491‐5p/TCF7 axis, which provides new insights into AS treatment strategies.

## Graphical abstract

The present study aimed to unravel the role of lncRNA LINC02381/miR‐491‐5p/TCF7 axis in regulating HUVECs injury. Results indicated that silencing of LINC02381 ameliorated HUVECs injury by regulating miR‐491‐5p/TCF7 axis, shedding new insights for AS treatment.

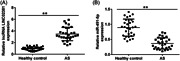

## 1 INTRODUCTION

According to the Global Burden of Disease institution, cardiovascular diseases have been the leading cause of mortality worldwide for more than 10 consecutive years.^1^ Atherosclerosis (AS) is the primary causative pathology of cardiovascular associated diseases,^2^ and is a systemic disease characterized by impaired lipid metabolism, intimal lipid deposition, atheromatous plaque formation, fibrous tissue proliferation, and vessel wall sclerosis in large and medium‐sized arteries.^3^ The clinical manifestations of AS result in heart disease,^4^ ischemic stroke,^5^ and peripheral arterial disease.^6^ Endothelial cell injury induced by oxidized low‐density lipoproteins (ox‐LDL) could contribute to the onset and development of AS. Human umbilical vein endothelial cells (HUVECs) have been widely utilized for in vitro studies of AS, with ox‐LDL‐induced HUVECs increasingly being used as an in vitro AS model. Previous studies have shown that the atherosclerotic process is closely associated with abnormal cellular proliferation and HUVEC apoptosis, which aggravate the progression of AS.^7^, ^8^ Additional evidence suggests that inflammatory responses exert a crucial role in the pathogenesis of AS.^9^ Inflammatory cytokines infiltrate the arterial wall, inducing foam cell formation and HUVEC apoptosis, leading to plaque growth, erosion, and rupture.^10^

Research into the association between noncoding RNAs (ncRNAs) and cardiovascular disease has recently gained attention,^11^ with several ncRNAs identified as biomarkers of different cardiovascular diseases.^12^ ncRNAs, including miRNAs, long noncoding RNAs (lncRNAs), and circRNAs, have the common characteristic of having transcriptional but no protein translation activity and play biological roles at the level of RNAs.^13^, ^14^ miRNAs are the major posttranscriptional gene regulators that regulate mRNA expression by inducing transcript degradation and translation repression.^15^ lncRNAs can bind to the target genes regulated by miRNAs, thereby competing with and consequently inhibiting the regulatory effects of miRNAs.^16^ Recent studies show that various types of ncRNAs modulate gene expression and play significant roles in the pathophysiology of AS. Li et al.^17^ reported that the expression of lncRNA taurine‐upregulated gene 1 (TUG1) was increased, whereas miR‐21 levels were decreased in serum samples of patients with AS and in the atherosclerotic plaques of ApoE^−/−^ mice. Yu et al.^18^ observed increased expression of lncRNA kcnq1ot1 and decreased expression of miR‐452‐3p in the aorta of mice with AS and in lipid‐loaded macrophages. Previous studies have reported decreased expression of miR‐491‐5p in atherosclerotic plaque tissues and serum samples from patients with AS.^19^, ^20^ miR‐491‐5p exerts a protective role in AS development by inhibiting the oxidative stress and inflammatory responses of THP‐1 macrophages via MMP‐9 and suppressing the cellular proliferation and migration of vascular smooth muscle cells.^19^, ^20^, ^21^, ^22^ A recent study revealed that miR‐491‐5p is associated with the protective effect of circ_0003204 gene silencing on ox‐LDL‐induced HUVECs injury.^23^ Thus, miR‐491‐5p may exert a protective role in AS development, however, the mechanisms underlying its actions remain to be identified. We used a bioinformatics approach to identify a potential binding site between LINC02381 and miR‐491‐5p. LINC02381 has been studied in several types of cancers and found to be involved in inflammatory diseases. In addition, LINC02381 has been suggested to act as a miRNA sponge for various miRNAs, including miR‐27b‐3p, miR‐1271‐5p, and miR‐21.^24^, ^25^, ^26^ However, the role of LINC02381 and its relationship with miR‐491‐5p in the pathophysiology of AS remain unclear. In this study, we investigated whether LINC02381 exerts a role in ox‐LDL‐induced endothelial injury by modulating miR‐491‐5p expression using in vitro experiments with HUVECs.

## 2 MATERIALS AND METHODS

### 2.1 Patient recruitment and sample collection

We recruited 30 participants with AS and 30 healthy control participants from the Fifth Hospital in Wuhan, China. Patients with AS were diagnosed using coronary angiography. Exclusion criteria were as follows: severe heart valve disease, cardiac function grade III or higher within 2 weeks after acute myocardial infarction, malignant tumors, severe hematologic diseases, and severe liver and renal failure. The characteristics of AS patients are displayed in Table 1. The study was accredited by the Ethics Committee of the Fifth Hospital in Wuhan (approval number: 2020020601012315) and adhered to the tenets of the Declaration of Helsinki. A written informed consent was signed and obtained from each participant before the study.

**Table 1:** The characteristics of participants with AS.

Parameters	Healthy control	AS patients	*p* Value
Male/female	15/15	15/15	–
Age (year)	51–68	52–71	>.05
Hypertension	4 (13.3%)	20 (66.7%)	<.05
Diabetes mellitus (%)	6 (20.0%)	7 (23.3%)	>.05
Current smoke (%)	8 (26.7%)	7 (23.3%)	>.05
TC (mg/dL)	190.35 ± 4.01	193.22 ± 3.45	>.05
TG (mg/dL)	121.62 ± 12.28	123.51 ± 13.79	>.05
HDL (mg/dL)	47.88 ± 4.33	46.32 ± 4.63	>.05
LDL (mg/dL)	115.81 ± 7.06	116.77 ± 6.59	>.05
DBP (mm Hg)	69.83 ± 4.06	81.83 ± 5.21	<.05
CRP (mg/L)	2.81 ± 1.24	9.83 ± 2.21	<.05
CIMT (mm)	0.55 ± 0.14	1.04 ± 0.11	<.05

Abbreviations: AS, atherosclerosis; CIMT, carotid intima‐media thickness; CRP, C‐reactive protein; DBP, diastolic blood pressure; HDL, high‐density lipoprotein; LDL, low‐density lipoprotein; TC, total cholesterol; TG, triglyceride.

After recruitment and fasting for 8 h, 5 mL serum samples were extracted from each participant and stored at −80°C until analysis.

### 2.2 Cell culture and ox‐LDL treatment

HUVECs were obtained from American Type Culture Collection and cultivated in Dulbecco's Modified Eagle Medium (Invitrogen; Thermo Fisher Scientific Inc.) at 37°C containing 5% CO_2_, 10% fetal bovine serum, and 1% 1% penicillin and streptomycin.

The cells were divided into control and ox‐LDL groups (0, 50, 100, and 150 μg/mL) and plated into 96‐well plates at a density of 4 × 10^4^ cells/mL.^27^ Cells in the ox‐LDL group were exposed to different concentrations of ox‐LDL for 24 h at 37°C with 5% CO_2_.

### 2.3 Cellular transfection

TCF7‐plasmid and its negative control (control‐plasmid, no. sc‐36617) were constructed and purchased from Santa Cruz Biotechnology. Control‐siRNAs, LINC02381‐siRNAs, miR‐491‐5p suppressor and its suppressor control, and miR‐491‐5p mimic and its mimic control were purchased from Guangzhou RiboBio Co. Ltd. Control‐siRNA, LINC02381‐siRNA, miR‐491‐5p inhibitor, inhibitor control, LINC02381‐siRNA + inhibitor control, LINC02381‐siRNA + miR‐491‐5p inhibitor, mimic control, miR‐491‐5p mimic, control‐plasmid, TCF7‐plasmid, miR‐491‐5p mimic + control‐plasmid, or miR‐491‐5p mimic + TCF7‐plasmid were transfected into HUVECs using Lipofectamine 2000 reagent (Invitrogen; Thermo Fisher Scientific Inc.), as per the manufacturer's protocol.

### 2.4 Reverse transcription‐quantitative polymerase chain reaction assay

Serum samples and cells were collected for RNA extraction using the TRIzol Kit (Takara). DNase I (Thermo Fisher Scientific Inc.) was added to the extracted RNA to digest the genomic DNA, followed by cDNA reverse transcription using the TaqMan RNA Kit (Invitrogen; Thermo Fisher Scientific Inc.). The polymerase chain reaction (PCR) was conducted on the ABI 7500 System (Applied Biosystems) with the SYBR Premix Kit (Thermo Fisher Scientific Inc.). Thermal cycles included initial denaturation at 95°C for 5 min, followed by 40 cycles at 95°C for 10 s and at 64°C for 20 s. Results were processed using the $2^{‐∆∆C_{T}}$ method^28^ for relative quantification of gene expression, with GAPDH and U6 genes as the internal references.^18^, ^29^, ^30^ Primer sequences used were as follows:

LINC02381 forward 5′‐CTGATGGCCACTCACGCTAT‐3′

reverse 5′‐GATCCGGAGGGAGAGCATTC‐3′^29^;

GAPDH forward 5′‐TCCTGTGGCATCCACGAAACT‐3′;

reverse 5′‐GAAGCATTTGCGGTGGACGAT‐3′^29^;

TCF7 forward 5′‐CTGGCTTCTACTCCCTGACCT‐3′;

reverse 5′‐ACCAGAACCTAGCATCAAGGA‐3′;

miR‐491‐5p forward 5′‐GGAGTGGGGAACCCTTCC‐3′;

reverse 5′‐GTGCAGGGTCCGAGGT‐3′^30^;

U6 forward 5′‐CTCGCTTCGGCAGCACA‐3′;

reverse 5′‐AACGCTTCACGAATTTGCGT‐3′.^30^

### 2.5 Enzyme‐linked immunosorbent assay assay

The expression levels of tumor necrosis factor‐alpha (TNF‐α), interleukin (IL)‐6, and IL‐1β in the cell supernatants were evaluated using their associated enzyme‐linked immunosorbent assay (ELISA) kits (TNF‐α, #7355; IL‐6, #8904; IL‐1β #8900; Cell Signaling Technology).^31^

### 2.6 Cell Counting Kit‐8 assay to evaluate cell viability

HUVECs in the logarithmic growth phase were harvested and counted after digestion with 0.25% trypsin in 5 × 10^4^ cells/mL cellular suspension samples. Approximately 100 μL of the cell suspension was plated in 96‐well plates and incubated at 37°C in a 5% CO_2_ incubator. At 0, 24, 48, and 72 h, 10 μL of Cell Counting Kit‐8 solution was supplemented into each well and incubated for another 2 h at room temperature. Eventually, an enzyme marker was used to measure the sample absorbances.^32^

### 2.7 Flow cytometry assay for cell apoptosis

The transfected cells were collected by cellular digestion using 0.25% trypsin without EDTA. Cells were removed into a centrifuge tube for centrifugation at 1000 × *g* for 5 min at 4°C. A binding buffer was used to resuspend the cells, which were diluted to a cellular concentration of 1 × 10^6^ cells/mL. Subsequently, 5 μL of Annexin V‐FITC and 5 μL of propidium iodide at a concentration of 20 μg/mL were added to each well and incubated for 15 min at 25°C in the dark. Stained HUVECs were measured using flow cytometry (BD Bioscience) and Kaluza Analysis (version 2.1.1.20653; Beckman Coulter Inc.).^33^

### 2.8 Western blot assay

After cell treatments, 100 μL of radioimmunoprecipitation assay buffer containing 1 μmol/L phenylmethanesulfonyl fluoride was added to the treated cells and incubated on ice for 20 min. The cell lysate was transferred to a microcentrifuge tube and centrifuged at 12,000 rpm for 10 min, and the supernatant was removed for protein concentration calculation using the BCA method. SDS‐gel electrophoresis was performed, and the gel was subsequently placed in the electrotransfer solution and equilibrated for 15 min. Protein samples were then transferred to polyvinylidene fluoride membranes using the wet transfer method. Membranes were blocked in 5% skim milk for 2 h and incubated with primary antibodies (cleaved‐caspase‐3, 1:1000, ab2302; caspase‐3, 1:1000, ab32351; GAPDH, 1:2000, ab9485; Abcam) overnight at 4°C. The membranes were then washed three times with TBST buffer at 25°C and incubated with secondary antibodies (goat anti‐rabbit IgG H&L [HRP] preadsorbed, 1:1000, ab7090; Abcam) for 2 h at 25°C. ECL solution was used for luminescence. The Western blot bands were scanned in grayscale using ImageJ software.^34^

### 2.9 Dual‐luciferase reporter assay

The whole length of LINC02381 and TCF7 was designed and obtained using PCR amplification and inserted in the psiCHECK‐2 vector to generate wild‐type LINC02381 (LINC02381‐WT) and wide‐type TCF7 (TCF7‐WT), whereas mutant LINC02381 (LINC02381‐MUT) and TCF7 (TCF7‐MUT) were engineered and purchased from GenePharma. The plasmids were incubated and transfected with miR‐491‐5p mimic or mimic control into HUVECs using Lipofectamine 2000 reagent (Invitrogen; Thermo Fisher Scientific Inc.), as per the manufacturer's instructions. After 24 h of transfection, Dual Luciferase Assay Kit was utilized to measure luciferase activity (Zeye Inc.).^35^

### 2.10 RNA pull‐down assay

Cells at a concentration of 1 × 10^7^ cells were collected and lysed using an ultrasonic processor for 3 min. The LINC02381 probe was cotreated with magnetic beads at 25°C for 2 h and incubated with the cell lysate overnight at 4°C. After washing with elution buffer, the RNA complex bound to the magnetic beads was eluted, the RNA was extracted, and samples underwent qRT‐PCR to evaluate the miR‐491‐5p expression.^36^

### 2.11 Statistical analysis

Statistical analysis was conducted using SPSS version 19.0, and statistical graphs were generated using GraphPad Prism 5.0. Data were expressed using means ± standard deviation. Unpaired, two‐tailed Student's *t*‐test was used for intergroup comparisons, and one‐way analysis of variance followed by Tukey's test was used for the comparison of multiple samples. For all analyses, a *p* < .05 indicated statistical significance.

## 3 RESULTS

### 3.1 Aberrant expressions of LINC02381 and miR‐491‐5p in serum samples from patients with AS

**Figure 1:**
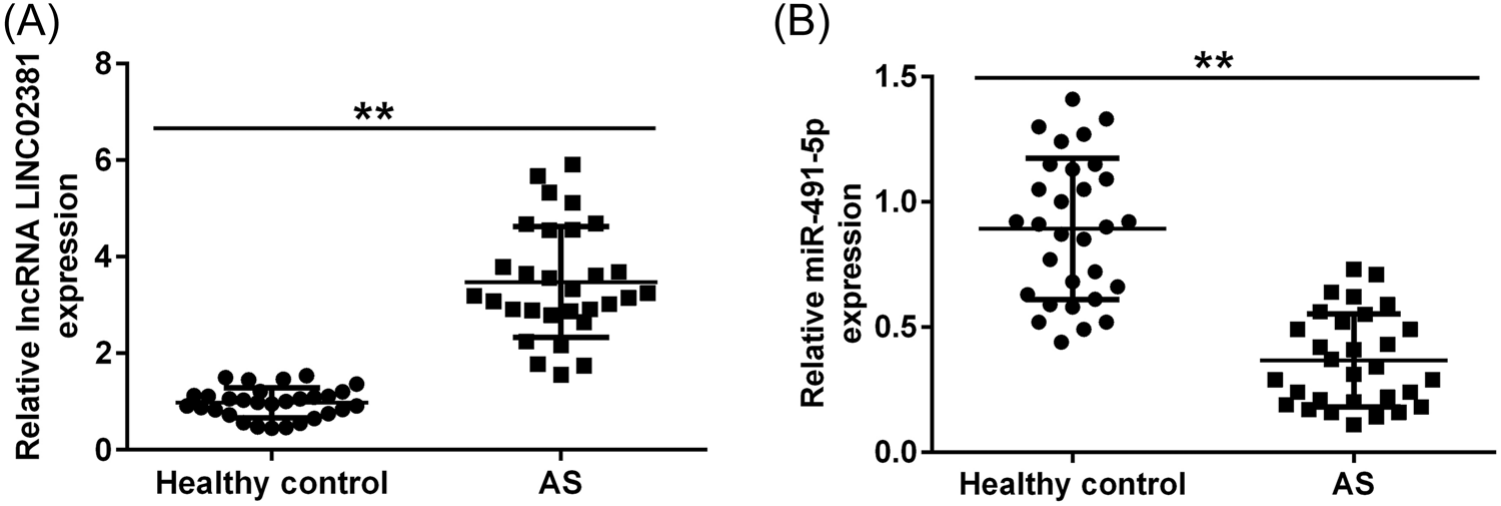
Expression of LINC02381 and miR‐491‐5p in serum samples of patients with atherosclerosis (AS) (*n* = 30). (A) LINC02381 levels detected by reverse transcription‐quantitative polymerase chain reaction (RT‐qPCR) in serum samples from 30 patients with AS and 30 healthy controls. (B) miR‐491‐5p levels detected by RT‐qPCR in serum samples from 30 patients with AS and 30 healthy controls. **p < .01 versus Healthy control.

We determined the expression levels of LINC02381 and miR‐491‐5p in serum samples from 30 participants with AS and 30 corresponding healthy participants using RT‐qPCR. As displayed in Figure 1A, LINC02381 levels were significantly elevated in participants with AS compared to healthy controls. Contrarily, miR‐491‐5p was significantly decreased in patients with AS in comparison with healthy participants (Figure 1B).

### 3.2 Targeted association between LINC02381 and miR‐491‐5p

**Figure 2:**
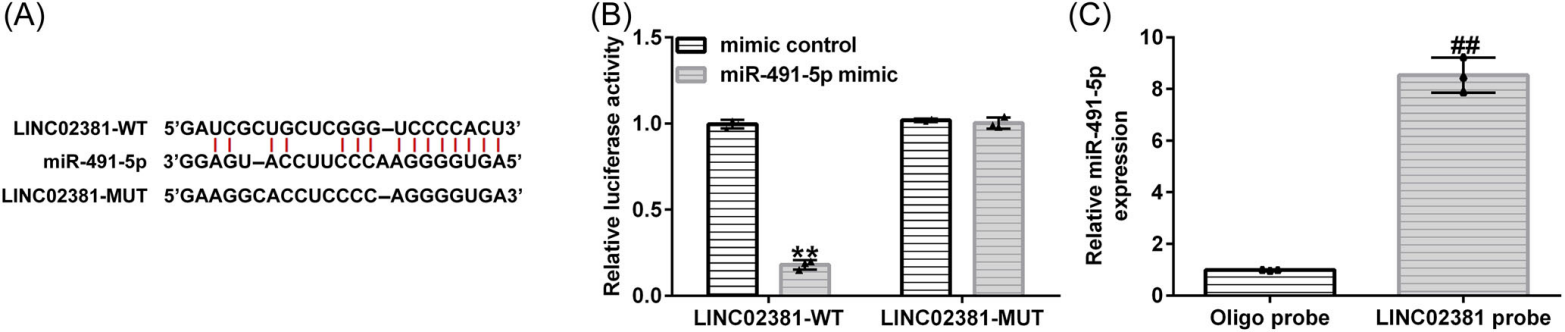
Targeted relationship between miR‐491‐5p and LINC02381. (A) starBase tool predicted the binding sites between miR‐491‐5p and LINC02381. (B) Dual‐luciferase reporter assay (*n* = 3). (C) RNA pull‐down assay (*n* = 3). **p < 0.01 versus mimic control; ^##^ *p* < 0.01 versus Oligo probe.

We confirmed the association between LINC02381 and miR‐491‐5p using dual‐luciferase reporter and RNA pull‐down assays. We used the starBase online platform to predict the putative binding sequences between LINC02381 and miR‐491‐5p (Figure 2A). The dual‐luciferase reporter assay verified that cotransfection with WT‐LINC02381 and miR‐491‐5p mimic prominently decreased luciferase activity compared to that with WT‐LINC02381 and mimic control (Figure 2B). Nevertheless, no prominent variations were found between the Mut‐LINC02381 transfection groups. Furthermore, the RNA pull‐down assay confirmed that miR‐491‐5p was abundantly enriched in the LINC02381 probe compared to the input group, suggesting that this probe can specifically bind with miR‐491‐5p (Figure 2C).

### 3.3 Effects of ox‐LDL treatment on LINC02381 and miR‐491‐5p expressions in HUVECs

**Figure 3:**
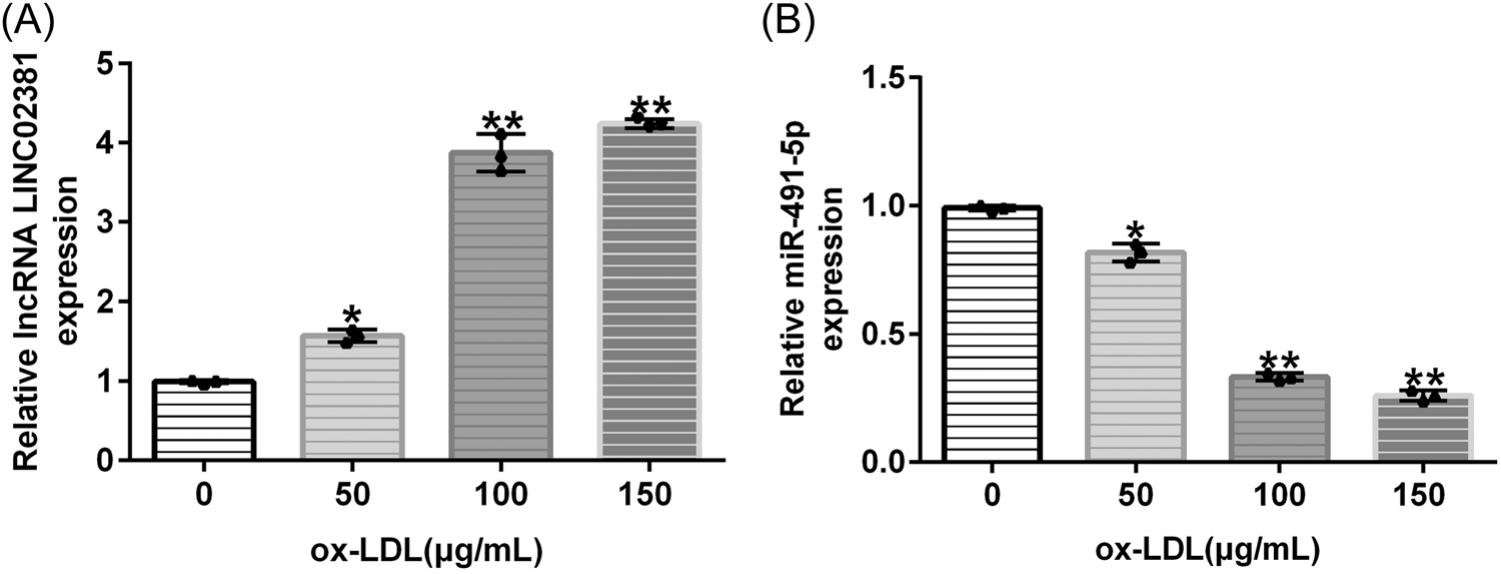
Effects of aberrant LINC02381 levels on miR‐491‐5p expression in human umbilical vein endothelial cells (HUVECs). HUVECs were exposed to different concentrations (0, 50, 100, and 150 μg/mL) of oxidized low‐density lipoprotein (ox‐LDL) for 24 h. (A) Expression of LINC02381 was determined using reverse transcription‐quantitative polymerase chain reaction (RT‐qPCR) assay (*n* = 3). (B) Expression of miR‐491‐5p was determined using RT‐qPCR assay (*n* = 3). *p < .05 versus Control (0 μg/mL ox‐LDL); **p < .01 versus Control (0 μg/mL ox‐LDL).

We investigated the effects of treatment with different doses of ox‐LDL for 24 h (0, 50, 100, and 150 μg/mL) on LINC02381 and miR‐491‐5p levels in HUVECs using RT‐qPCR analysis. We observed that LINC02381 levels gradually increased with increasing doses of ox‐LDL (Figure 3A), whereas miR‐491‐5p levels were downregulated with increasing doses of ox‐LDL in a dose‐independent manner (Figure 3B).

### 3.4 Effects of LINC02381 on miR‐491‐5p in HUVECs

**Figure 4:**
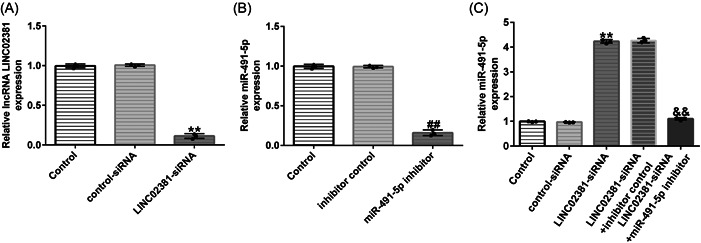
Regulatory mechanisms of LINC02381 on miR‐491‐5p expression in human umbilical vein endothelial cells (HUVECs). (A) Transfection efficiency of LINC02381‐siRNA in HUVECs (*n* = 3). (B) Transfection efficiency of miR‐491‐5p inhibitor in HUVECs (*n* = 3). (C) Expression of miR‐491‐5p in HUVECs after transfection with LINC02381‐siRNA and/or miR‐491‐5p inhibitor (*n* = 3). **p < .01 versus Control‐siRNA; ^##^ *p* < .01 versus inhibitor control; ^&&^ *p* < .01 versus LINC02381‐siRNA + inhibitor control.

We transfected LINC02381 and miR‐491‐5p siRNA and inhibitor, respectively, in HUVECs to elucidate the relationship between them. As demonstrated in Figure 4A, LINC02381 expression was significantly downregulated after transfection in the LINC02381‐siRNA group, in comparison with the control‐siRNA group. Furthermore, the transfection efficacy experiment in Figure 4B illustrated that the miR‐491‐5p inhibitor successfully downregulated miR‐491‐5p expression compared with the inhibitor control group. Finally, LINC02381‐siRNA significantly upregulated miR‐491‐5p expression in HUVECs, whereas a partial enhancement was observed after cotransfection with the miR‐491‐5p inhibitor (Figure 4C).

### 3.5 LINC02381 knockdown is protective against ox‐LDL‐induced HUVEC injury due to miR‐491‐5p upregulation

**Figure 5:**
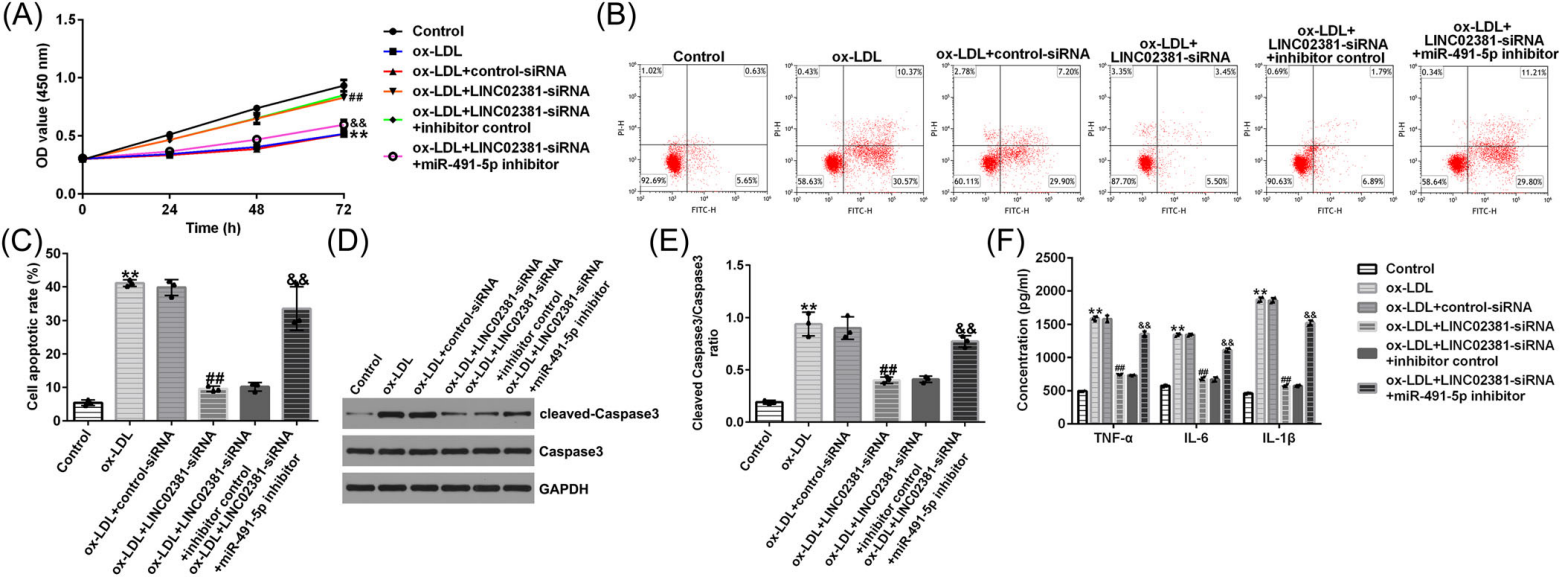
LINC02381 knockdown alleviated oxidized low‐density lipoprotein (ox‐LDL)‐induced human umbilical vein endothelial cell injury by regulating miR‐491‐5p. (A) Cell Counting Kit‐8 assay determined cell viability. (B, C) Cell apoptosis was quantified using flow cytometry assay. (D) Western blot assay detected cleaved‐caspase‐3 and caspase‐3 expressions. (E) Quantified results of cleaved‐caspase‐3/caspase‐3. (F) tumor necrosis factor‐α, interleukin‐6 (IL‐6), and IL‐1β levels were detected using enzyme‐linked immunosorbent assay assay. *n* = 3; **p < .01 versus Control; ^##^ *p* < .01 versus ox‐LDL + control‐siRNA; ^&&^ *p* < .01 versus ox‐LDL + LINC02381‐siRNA + inhibitor control.

We used LINC02381‐siRNA to investigate the role of LINC02381 knockdown in ox‐LDL‐induced HUVEC injury. Ox‐LDL‐induced endothelial cell injury, evidenced by reduced cell proliferation and enhanced cell apoptosis and inflammatory response, contributes to the onset and development of AS. Caspase‐3 is the most important terminal cleavage enzyme in the process of apoptosis. Since caspase‐3 is activated by proteolytic cleavage, we performed a Western blot assay to detect total caspase‐3 and cleaved‐caspase‐3 levels. We detected the levels of the proinflammatory cytokines, TNF‐α, IL‐6, and IL‐1β, which are involved in accelerating AS progression, using ELISA. We observed that treatment with ox‐LDL significantly impaired HUVEC cell viability and increased apoptosis, cleaved‐caspase‐3 levels, and proinflammatory cytokine secretion, including TNF‐α, IL‐6, and IL‐1β (Figure 5A–F). Moreover, knockdown of LINC02381 also resulted in ox‐LDL‐induced cell viability reduction and apoptosis and inflammation response enhancement compared with the ox‐LDL + control‐siRNA group. Moreover, the therapeutic role of LINC02381 knockdown on ox‐LDL‐induced HUVEC injury could be partially restored with miR‐491‐5p inhibitor cotransfection.

### 3.6 TCF7 is a downstream target and negative regulator of miR‐491‐5p

**Figure 6:**
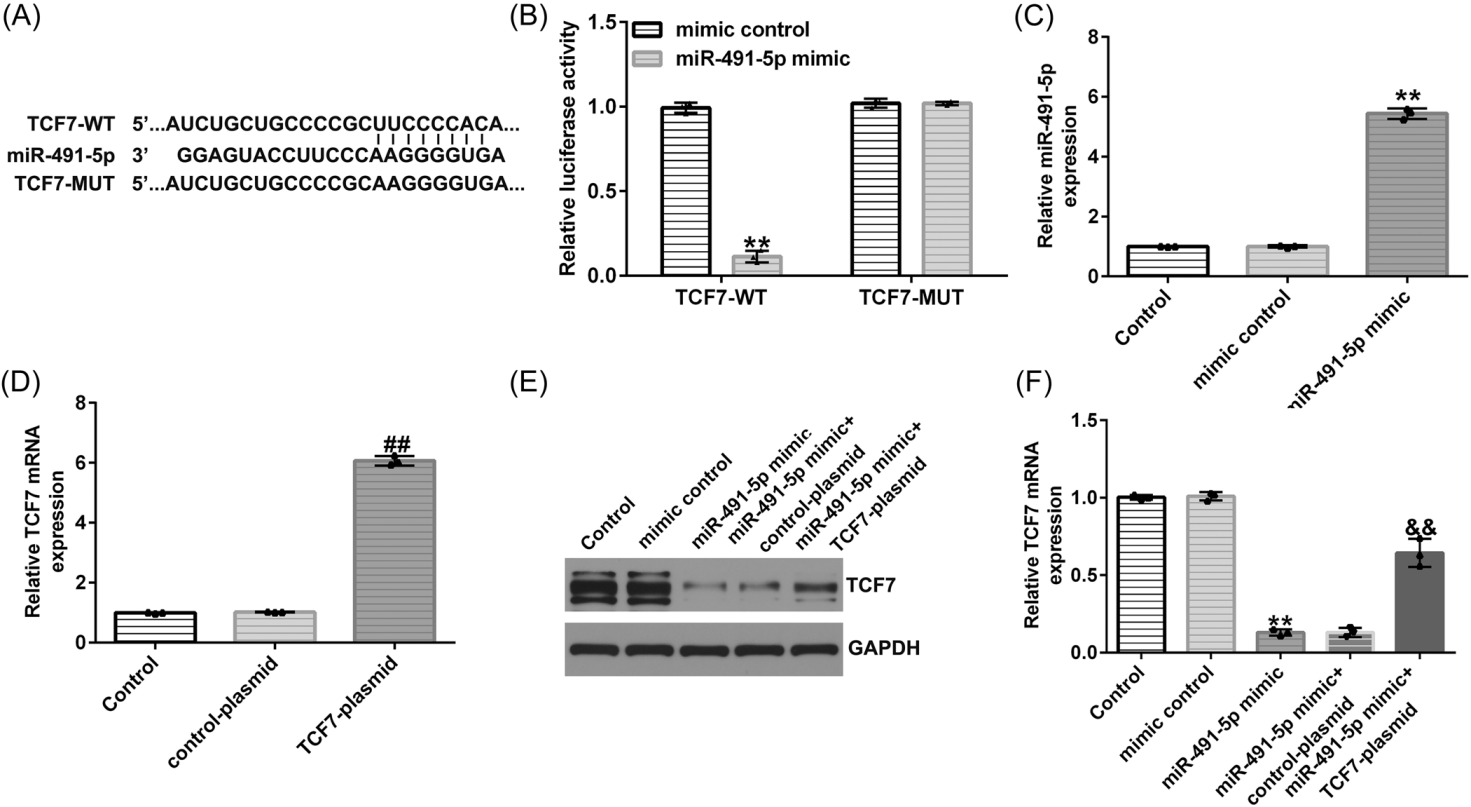
Targeted relationship between TCF7 and miR‐491‐5p. (A) TargetScan tool predicted the binding sequences between TCF7 and miR‐491‐5p. (B) Dual‐luciferase reporter assay confirmed the targeted relationship between miR‐491‐5p and TCF7 (*n* = 3). (C) Transfection efficacy of miR‐491‐5p mimic in human umbilical vein endothelial cells (HUVECs). (D) Transfection efficacy of TCF7 overexpression in HUVECs (*n* = 3). (E) mRNA expression of TCF7 after cotransfection with TCF7 plasmid and miR‐491‐5p mimic (*n* = 3). (F) Protein expression of TCF7 after cotransfection with TCF7‐plasmid and miR‐491‐5p mimic (*n* = 3). **p < .01 versus mimic control; ^##^ *p* < .01 versus Control plasmid; ^&&^ *p* < .01 versus miR‐491‐5p mimic + control‐plasmid.

We used multiple prediction software including PITA, miRmap, microT, and TargetScan version 7.2 (https://www.targetscan.org/vert_80/) to predict the potential targets of miR‐491‐5p. According to TargetScan software prediction results, miR‐491‐5p has hundreds of target genes including TCF7, which is reported to be significantly upregulated in atherosclerotic patient serum as well as in ox‐LDL‐induced HUVECs and is involved in the regulation of ox‐LDL‐induced HUVEC injury.^36^ Thus, we hypothesized that miR‐491‐5p may play a role in AS by regulating the expression of TCF7. Therefore, we selected TCF7 for further analysis. The putative binding sequences between miR‐491‐5p and TCF7 are presented in Figure 6A. The dual‐luciferase reporter assay was used to verify if cotransfection with WT‐TCF7 and miR‐491‐5p mimic reduced luciferase activity compared to that with WT‐TCF7 and the mimic control (Figure 6B). We did not observe any pronounced variation in the Mut‐TCF7 transfection groups. We further verified the regulatory mechanisms between miR‐491‐5p and TCF7 using rescue experiments. As shown in Figure 6C, miR‐491‐5p mimic significantly increased miR‐491‐5p expression levels in HUVECs. Meanwhile, transfection with TCF7‐plasmid increased TCF7 levels in HUVECs, suggesting a successful transfection (Figure 6D). In addition, RT‐qPCR and Western blotting assays revealed that the miR‐491‐5p mimic significantly suppressed mRNA and protein levels of TCF7, which was reversed with cotransfection of TCF7 plasmid (Figure 6E,F).

### 3.7 Expression of TCF7 in serum samples from patients with AS and in ox‐LDL‐induced HUVECs

**Figure 7:**
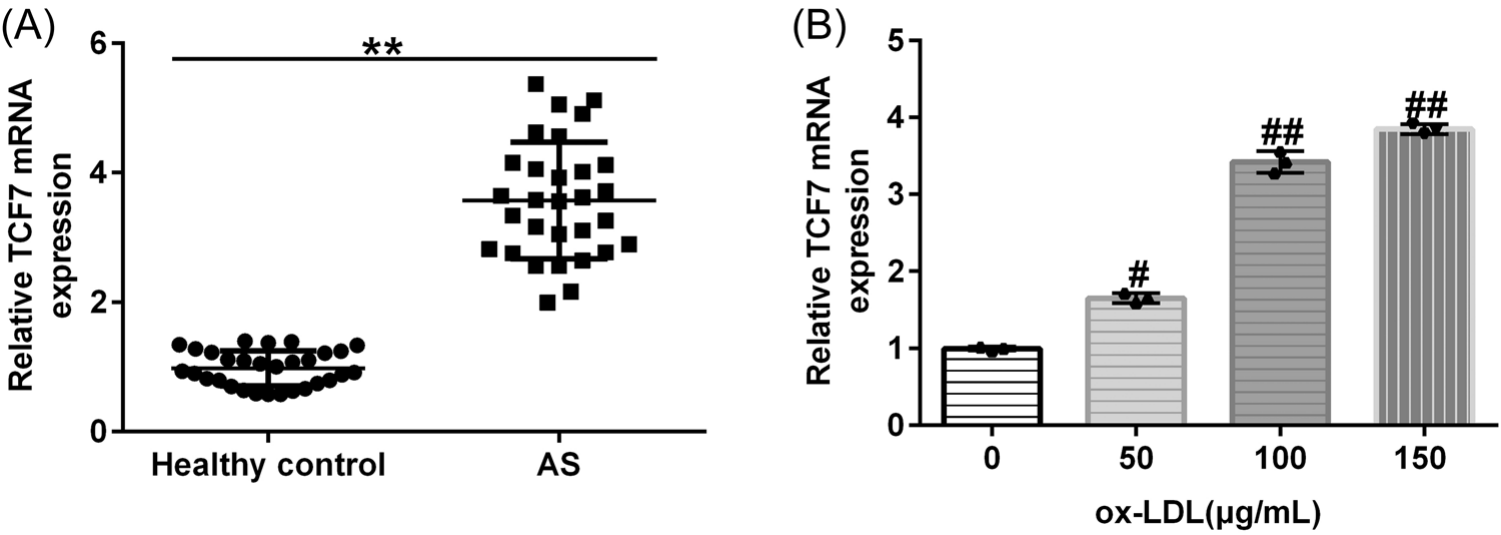
Expression of TCF7 mRNA in serum samples of patients with atherosclerosis (AS) and in oxidized low‐density lipoprotein (ox‐LDL)‐induced human umbilical vein endothelial cells (HUVECs). (A) TCF7 mRNA levels detected by reverse transcription‐quantitative polymerase chain reaction (RT‐qPCR) in serum samples from 30 patients with AS and 30 healthy controls (*n* = 30). (B) TCF7 mRNA levels detected by RT‐qPCR in HUVECs exposed to different concentrations (0, 50, 100, and 150 μg/mL) of ox‐LDL for 24 h (*n* = 3). **p < .01 versus Healthy control; ^##^ *p* < .01 versus Control (0 μg/mL ox‐LDL).

We determined the expression of TCF7 in serum samples from 30 participants with AS and in the ox‐LDL‐induced HUVECs model using RT‐qPCR. As displayed in Figure 7A, TCF7 mRNA levels were significantly upregulated in patients with AS compared to healthy controls. Moreover, the mRNA levels of TCF7 were also increased in ox‐LDL‐induced HUVECs in comparison with the control group (Figure 7B).

### 3.8 miR‐491‐5p alleviates ox‐LDL‐triggered‐HUVEC injury by regulating TCF7 expression

**Figure 8:**
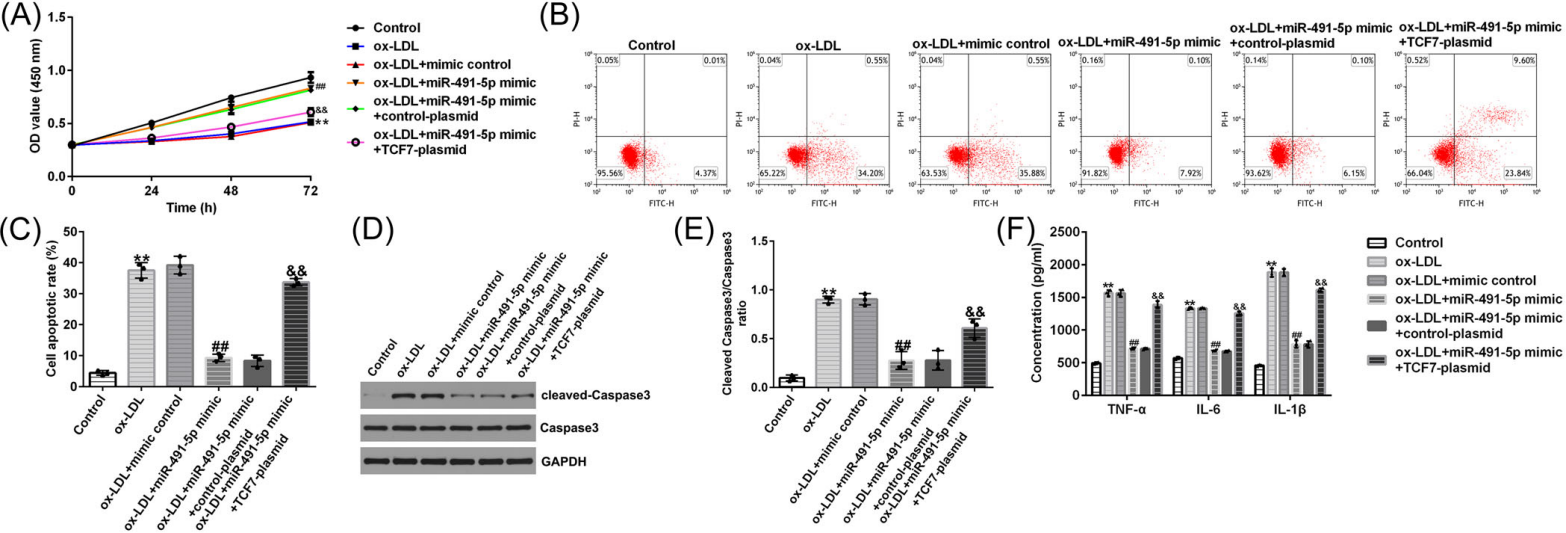
miR‐491‐5p protected against oxidized low‐density lipoprotein (ox‐LDL)‐induced human umbilical vein endothelial cell (HUVEC) injury by inhibiting TCF7 expression. HUVECs cells were exposed to 100 μg/mL ox‐LDL for 24 h and transfected with miR‐491‐5p mimic and/or TCF7 plasmid. (A) Cell viability was evaluated using Cell Counting Kit‐8 assay. (B, C) Cell apoptosis was determined using flow cytometry analysis. (D) Protein expressions of cleaved‐caspase‐3 and caspase‐3 were confirmed by Western blot assay. (E) Cleaved‐caspase‐3/caspase‐3 ratio was quantified. (F) Tumor necrosis factor‐α, interleukin‐6 (IL‐6), and IL‐1β levels were detected using enzyme‐linked immunosorbent assay assay. *n* = 3; **p < .01 versus Control; ^##^ *p* < .01 versus ox‐LDL + mimic control; ^&&^ *p* < .01 versus ox‐LDL + miR‐491‐5p mimic + control‐plasmid.

We cotransfected ox‐LDL‐treated HUVECs with miR‐491‐5p mimic and TCF7‐plasmid to investigate the effects of miR‐491‐5p and TCF7 on HUVEC injury by measuring cellular viability, apoptosis, and inflammatory response. As displayed in Figure 8A, ox‐LDL‐triggered reduction in cell viability was suppressed with the miR‐491‐5p mimic; however, this protective effect was antagonized by TCF7 overexpression. Moreover, ox‐LDL‐induced cellular apoptosis and elevated cleaved‐caspase‐3 levels were decreased with miR‐491‐5p overexpression (Figure 8B–E), whereas the reduction of cell apoptosis by miR‐491‐5p mimic was counteracted with the TCF7‐plasmid (Figure 8B–E). Furthermore, although ox‐LDL‐induced inflammatory response (TNF‐α, IL‐6, and IL‐1β) was inhibited by the miR‐491‐5p mimic (Figure 8F), these effects were reversed by treatment with TCF7 plasmid.

## 4 DISCUSSION

AS is a major contributor to coronary artery disease, stroke, cerebral infarction, and peripheral vascular disease, making it one of the most prevalent signs of global morbidity and mortality.^37^ Several factors have been reported to induce AS, and recent research has identified new causes of AS, including endothelial cell damage,^38^ lipid metabolism,^39^ intestinal microbiota dysbiosis,^40^ and *Chlamydia pneumoniae*.^41^ Treatment with ox‐LDL stimulates vascular cells to secrete inflammatory molecules and accumulate monocytes and T cells in the arterial wall.^42^ As monocytes in the vessel wall are precursors to lipid macrophages, they establish fatty streaks, which are an anatomical feature of early AS.^43^, ^44^ In this study, we successfully established an in vitro model of AS using HUVECs treated with ox‐LDL for 24 h, which resulted in significantly decreased cell viability and enhanced cellular apoptosis and inflammation, consistent with previous studies.^45^, ^46^

Acting as regulatory noncoding RNAs, lncRNAs have transcript lengths >200 nt that have no protein translation functions. lncRNAs were initially considered to be genomic transcriptional junk without any biological functions. However, recent reports have demonstrated that lncRNAs are strongly correlated with cardiovascular diseases, including AS. A study reported significant downregulation of the lncRNA, FAF, in patients with coronary heart disease, revealing a negative correlation with independent risk factors of coronary heart disease.^47^ Feng et al.^48^ similarly reported that lncRNA DCRF knockdown promoted cardiac function, suppressed cellular autophagy in cardiomyocytes, and consequently alleviated diabetic cardiomyopathy. Wu et al.^49^ identified six aberrantly expressed lncRNAs in AS using RNA sequencing. Furthermore, differentially expressed lncRNAs could identify underlying novel targets for the therapeutic diagnosis and treatment of AS. For instance, Bai et al.^50^ utilized microarray profiling analysis and identified 236 differentially expressed lncRNAs in AS, contributing to the diagnosis and treatment of AS. Meng et al.^51^ illustrated the clinical significance of the lncRNA, APPAT, in the diagnosis and progression of AS. Notably, LINC02381 was a newly identified tumor‐promoting lncRNA that was observed in various tumorigenesis processes, including osteosarcomas,^52^ gliomas,^53^ and cervical cancer.^29^ In contrast, LINC02381 expression was reduced in gastric cancers^54^ and colorectal carcinoma.^55^ In addition, LINC02381 was significantly upregulated in chronic autoimmune inflammatory diseases, accelerating rheumatoid arthritis development.^56^ In our study, we observed significantly increased levels of LINC02381 in AS serum samples and in ox‐LDL‐treated HUVECs. LINC02381 knockdown resulted in increased cell viability and decreased apoptosis and proinflammatory cytokines in ox‐LDL‐treated HUVECs. Thus, LINC02381 downregulation is protective against ox‐LDL‐induced injury in HUVECs, thereby alleviating AS progression.

miRNAs are a class of endogenous ncRNAs approximately 22 nt in length. They are highly evolutionarily conserved and regulate gene expression at the posttranscriptional level by binding to the 3′‐untranslated region (3′‐UTR) of target genes to degrade their mRNA or inhibit their translation.^57^ Several reports have demonstrated the role and biological significance of miR‐491‐5p in diabetes and AS. Sidorkiewicz et al.^58^ demonstrated that miR‐491‐5p was a putative diagnostic biomarker for type 2 diabetes mellitus, with an area under the curve of 94.0%. Another study illustrated that miR‐491‐5p was decreased in the tissues and plasma samples of participants with AS, indicating its protective role.^19^ In our study, dual‐luciferase reporter, RNA pull‐down, and rescue experiments confirmed the targeted association between LINC02381 and miR‐491‐5p. Moreover, we found prominently decreased miR‐491‐5p levels in AS serum samples and in ox‐LDL‐induced HUVECs. Overexpression of miR‐491‐5p was protective against the ox‐LDL‐induced HUVEC injury model by improving cellular viability and suppressing cellular apoptosis and inflammation response. Moreover, our findings demonstrated that LINC02381 knockdown could mitigate ox‐LDL‐triggered HUVEC injury by upregulating miR‐491‐5p. This is further supported by the finding that lncRNAs act as sponges to regulate miRNAs.^59^ Since miRNAs regulate cellular processes by binding to the 3′‐UTR of target genes, the TargetScan tool was utilized to predict the associated targets of miR‐491‐5p. Among them, TCF7 was screened as a candidate, and the targeted relationship between miR‐491‐5p and TCF7 was validated using the dual‐luciferase reporter assay. TCF7 has been reported to be involved in cardiovascular diseases, such as cardiac hypertrophy and acute coronary syndrome.^60^, ^61^, ^62^ A recent study showed that TCF7 is highly expressed in immune cells on atherosclerotic plaques, and regulates inflammatory signaling via the NFκB/AKT/STAT1 pathway.^63^ Moreover, in a previous study, TCF7 was demonstrated to be overexpressed in AS, in which TCF7 knockdown was protective against AS.^36^ Similarly, in our study, we found that TCF7 mRNA levels were significantly upregulated in patients with AS, and the rescue experiments verified that the protective effects of miR‐491‐5p on AS progression could be partially suppressed by overexpressing TCF7.

There were some limitations in our experimental design and other aspects. For instance, the role of LINC02381 in ox‐LDL‐induced endothelial cell injury was not analyzed in AS animal models. Besides, whether the TCF7 gene is associated with the effect of LINC02381 on the ox‐LDL‐induced HUVECs injury remains to be clarified. Moreover, whether LINC02381 plays a role in predicting the prognosis of AS patients require further analysis. These issues will be addressed in our future studies.

## 5 CONCLUSION

LINC02381 knockdown enhanced cellular proliferation and suppressed the apoptosis and inflammatory responses of ox‐LDL‐treated HUVECs by regulating the miR‐491‐5p/TCF7 axis, consequently mitigating AS progression. Therefore, LINC02381 could be developed as a novel clinical target for AS therapy.

## AUTHOR CONTRIBUTIONS

Xizheng Zhu contributed to the study design, data collection, statistical analysis, data interpretation, and manuscript preparation. Hui Xu contributed to data collection and statistical analysis. Beijia Chen contributed to data collection, statistical analysis, and manuscript preparation. All authors read and approved the final manuscript.

## CONFLICTS OF INTEREST STATEMENT

The authors declare no conflict of interest.

## Data Availability

Datasets used and/or analyzed during the current study are available from the corresponding author on reasonable request.
